# Possible role of hemichannels in cancer

**DOI:** 10.3389/fphys.2014.00237

**Published:** 2014-06-27

**Authors:** Kurt A. Schalper, Daniel Carvajal-Hausdorf, Mauricio P. Oyarzo

**Affiliations:** ^1^Servicio Anatomía Patológica, Clínica Alemana de Santiago, Facultad de Medicina Clinica Alemana Universidad del DesarrolloSantiago, Chile; ^2^Department of Pathology, Yale School of MedicineNew Haven, CT, USA

**Keywords:** hemichannels, connexins, pannexins, cancer

## Abstract

In humans, connexins (Cxs) and pannexins (Panxs) are the building blocks of hemichannels. These proteins are frequently altered in neoplastic cells and have traditionally been considered as tumor suppressors. Alteration of Cxs and Panxs in cancer cells can be due to genetic, epigenetic and post-transcriptional/post-translational events. Activated hemichannels mediate the diffusional membrane transport of ions and small signaling molecules. In the last decade hemichannels have been shown to participate in diverse cell processes including the modulation of cell proliferation and survival. However, their possible role in tumor growth and expansion remains largely unexplored. Herein, we hypothesize about the possible role of hemichannels in carcinogenesis and tumor progression. To support this theory, we summarize the evidence regarding the involvement of hemichannels in cell proliferation and migration, as well as their possible role in the anti-tumor immune responses. In addition, we discuss the evidence linking hemichannels with cancer in diverse models and comment on the current technical limitations for their study.

## Overview of mammalian hemichannels and methodological considerations

Multicellular organisms have evolved with sophisticated routes for cell-to-cell communication. Such interactions are multidimensional (e.g., they occur between different types of cells, in diverse spatial orientation/direction, environmental conditions and time frames) and create signaling circuits within tissues, ultimately allowing for a more efficient use of resources and coordination of responses. In addition to a myriad of membrane receptors, channels and transporters, mammalian cells are equipped with relatively large, low-resistance proteinaceous conduits to transmit signaling molecules such as ions (e.g., Na^+^ and K^+^), second messengers (e.g., Ca^2+^ and phosphatidylinositol 1,4,5-trisphosphate [IP_3_]), nucleotides (e.g., cAMP, ADP, and ATP) and metabolites (e.g., glucose, adenosine, glutamate and glutathione) across electrochemical gradients. Such structures correspond to aqueous pores formed by hexamerization of subunits of two topologically related vertebrate protein families commonly referred to as “gap junction-type proteins”: connexins (Cxs) and pannexins (Panxs). In humans, there are 21 different Cxs genes with a wide genomic distribution and 3 genes encoding Panxs located in chromosomes 11 (Panx1 and 3) and 22 (Panx2) (Baranova et al., 2004; Söhl and Willecke, 2004). Cxs and Panxs show no sequence homology and there is no evidence supporting formation of mixed Cxs/Panxs based channels. Alternative splicing variants and pseudogenes for some of these transcripts have been described in humans, but their biological relevance is uncertain. Paradoxically (and for historical reasons), when such channels are recognized as single structures they are termed “hemichannels” or “connexons,” although they really are whole, functional cell membrane channels allowing the passage of relatively small solutes up to ~1.2 kDa. When two of such channels/connexons, each from one adjacent cell, converge to areas of intercellular membrane contact and serially dock allowing for continuous communication of cytosols, they are termed intercellular gap junction channels (Sáez et al., 2013). In a conservative view, hemichannels have been considered to remain preferentially closed in resting conditions and to serve mainly as structural precursors of gap junction channels through lateral diffusion and clustering after membrane insertion (Gaietta et al., 2002). However, independent site-specific and cytoskeleton-dependent membrane targeting routes for hemichannels, either to areas of cell-cell junctions or to unopposed membrane clusters, have been described (Shaw et al., 2007). The relevance of Cx-based hemichannels and intercellular channels in physiology has become evident by the demonstration of disease phenotypes associated with Cxs mutations affecting protein conformation, turnover and channels function (reviewed in Schalper et al., 2009; Pfenniger et al., 2011).

Diverse structural and functional factors limit the study Cx and Panx hemichannels and intercellular gap junction channels in biological systems. The first major challenge is clear discrimination between hemichannels and intercellular channels. Hemichannels and intercellular channels formed by the same Cx or Panx usually coexist in cells and show overlapping permeability to ions/molecules and a similar (not highly specific) pharmacological sensitivity (Retamal et al., 2007a; Giaume and Theis, 2010; Fiori et al., 2012). In addition, hemichannels and gap junction channels formed by most studied Cxs and Panxs share a common amino acid sequence, secondary structure and transcriptional/post-transcriptional regulation (Sáez et al., 2013; Riquelme et al., 2013a). Therefore, molecular methods or conventional antibody-based detection are usually limited to study the levels, location, stoichiometry and participation of each channel type in any given response. In addition, the difficulties to recapitulate *in vitro* the complex multi-cellular/multidimensional tissue conditions have limited a clear dissection of the relative contribution of each channel type to various physiological and pathological processes. To overcome some of the aforementioned limitations, mimetic peptides and antibodies targeting specific regions at the extracellular (docking) domains have been used to allow structure-specific recognition/blockade of hemichannels (recently reviewed in Riquelme et al., 2013a).

The second major problem is discriminating between the contribution of Cx and Panx-based channels to any given response. Channels and hemichannels formed by Cxs or Panxs have functional, pharmacological similarities and overlapping expression patterns. In particular, Panxs have been shown to have glycosylation sites on the extracellular loop and a high glycosylation level could preclude the serial docking of Panx hemichannels (Boassa et al., 2008; Peñuela et al., 2013). This led to the notion that Panxs form exclusively hemichannels and not intercellular gap junction channels (Sosinsky et al., 2011). However, recent studies confirmed the early findings by Bruzzone et al. (2003) showing that at least Panx1 and 3 can form functional intercellular gap junction channels with independent properties (Sahu et al., 2014). Future studies exploring diverse cell/tissues and various experimental conditions will be required to support and extend this concept. Further details on the transcriptional regulation of Cx and Panx genes, structural and functional characteristics of Cx- and Panx-based channels, post-translational modifications, pharmacological properties and methodological considerations are discussed in comprehensive reviews published elsewhere by our group and by others (Goodenough and Paul, 2003; Sáez et al., 2013, 2010; Baranova et al., 2004; Söhl and Willecke, 2004; Panchin, 2005; Schalper et al., 2008b; Giaume and Theis, 2010; Kar et al., 2012; D'Hondt et al., 2013; Peñuela et al., 2013).

The aforementioned methodological limitations for the study of hemichannels both *in vitro* and *in vivo* and the possible “contamination” of results by additional yet anonymous transmembrane routes have pointed out possible flaws in the interpretation of correlative dyes/molecules uptake or release and electrophysiological studies demonstrating hemichannel existence and functions (Spray et al., 2006). However, the evidence on intercellular gap junction channels also largely relies on comparable correlative expression/function studies using dye transfer and electrophysiological experiments combined with pharmacological blockade. Direct intercellular communication pathways different from gap junction channels termed intercellular nanotubes have recently been described (reviewed in Sherer, 2013) and should be considered in the interpretation of gap junction studies. In addition, the intercellular transfer of regulatory molecules in specialized small bi-layered membranous vesicles termed exosomes (or ectosomes) could also contribute to some of the responses attributed exclusively to gap junction channels, particularly in the central nervous system (Kalani et al., 2014), immune system (Hwang, 2013) and cancer cells (Azmi et al., 2013). Channel-independent functions of Cxs and Panxs have also been well described and add difficulty to the interpretation of results (Vinken et al., 2012). Most studies evaluating the functions and properties of intercellular channels in various conditions have not simultaneously addressed possible changes in hemichannel functions. Thus, a comparable degree of skepticism should exist on the notion of the exclusive involvement of intercellular channels in many studies correlating Cx and Panx expression with certain responses or phenotypes. Finally, visual localization of hemichannels and gap junction channels has been performed largely using antibodies, some of which have not been thoroughly validated regarding their specificity, optimal titration/dynamic range, reproducibility and stability over time. The lack of specificity and reproducibility of commercial and in-house established antibodies represents a common flaw in biomedical research (Bordeaux et al., 2010). In this regard, personal experience working in the gap junction field, as well as reports by other authors have highlighted possible limitations and conflicting results using presumably specific antibodies (Yahalom et al., 1991; Coppen et al., 2002; Shurman et al., 2005; Brisset et al., 2009; Cone et al., 2013). Moreover, the relative contribution of technical error and biological variations in antibody-based results (e.g., diverse protein folding, sample fixation, antigen retrieval, blocking solutions, alternative transcripts, and posttranslational modifications) are not easy to discern. Careful validation of reagents and, more important, systematic reporting of antibodies lacking specificity/reproducibility could help to overcome this complex scenario.

## Gap junction proteins and cancer

After the seminal work by Drs. Werner Loewenstein and Yoshinobu Kanno showing impaired intercellular electrical coupling in chemically-induced and xenografted rat hepatocarcinomas (Loewenstein and Kanno, 1966, 1967), the role of gap junctions in cancer cells has been a subject of interest for the last five decades. A considerable number of articles on this subject have been published and the literature has been periodically reviewed (see Loewenstein, 1980; Trosko et al., 1983; Kanno, 1985; Yamasaki, 1990; Mesnil and Yamasaki, 1993; Mesnil et al., 1995; Trosko and Ruch, 1998; Mesnil, 2002; Naus, 2002; Mesnil et al., 2005; Cronier et al., 2009; Kandouz and Batist, 2010; Naus and Laird, 2010). In general, cancer cells from various types and after diverse experimental challenges have been shown to have lower expression of their native gap junction proteins than non-tumor samples. Also, highly proliferative tumor cells show atypical (e.g., predominantly cytoplasmic) expression of these proteins and impaired gap junctional intercellular communication. Restoration of the gap junction-type proteins and/or intercellular communication is frequently associated with reduced cell proliferation, which led to the broad concept that Cxs and Panxs were tumor suppressors. However, emerging data on the role of these proteins in tumor cell migration and metastasis have challenged this paradigm and pointed to situations in which these proteins could actually favor cancer progression (Cronier et al., 2009; Kandouz and Batist, 2010; Naus and Laird, 2010). Unfortunately, little of the knowledge on this subject has been translated to cancer medicine and the possible role of hemichannels in carcinogenesis and tumor progression remains largely unexplored. Previous studies on hemichannels have used transformed cell models. However, key experiments addressing the functions and impact of hemichannels in cancer cells have not yet been communicated and include: (i) detailed characterization of the presence and relative abundance of hemichannels in cancer cells; (ii) evaluation of hemichannel-mediated molecule uptake/release in tumor cells as compared to non-tumor cells; (iii) functional consequences of hemichannels activation and blockade in tumor cells and neoplasms and (iv) prognostic and predictive value of hemichannels expression/activation in human malignancies.

In this article, we hypothesize that functional hemichannels can be present in tumor cells (at least of some tumor subtypes or tumor cell subpopulations) and they could alter tumor cell proliferation and disease progression through the transmembrane exchange of signaling solutes such as nucleotides and Ca^2+^. To test this theory, we review the literature regarding the involvement of hemichannels in cancer-related processes such as cell proliferation/death and cell migration; and revisit some of the gap junction/cancer studies from a “hemichannel-centric” perspective. We also speculate on the possible role of hemichannels in cancer progression and as a clinically useful biomarker.

## Regulation of Cx and Panx expression in human cancer

Most tumor cells harbor genetic and epigenetic defects that lead to altered protein expression and signaling. The presence of gene amplification, rearrangements and acquisition of the so-called “driver” activating mutations are hallmarks of many clinically actionable oncogenes including HER2, EGFR, KRAS, c-KIT, and ALK. In addition, malignant cells frequently downregulate or silence transcripts associated with anti-tumor characteristics (e.g., tumor suppressor genes) such as P53, RB, and PTEN. Silencing of such alleles can occur by gene deletion, inactivating mutations or by epigenetic alterations (e.g., promoter hypermethylation and histone modifications). Finally, tumor cells can also alter the expression of transcripts post-transcriptionally by microRNA-mediated silencing or using the more recently described long non-coding RNAs (lncRNAs) system (Hauptman and Glavac, 2013).

Cxs and Panxs have been frequently shown to display altered expression in human tumors and cancer cell lines. Diverse studies have addressed the underlying mechanisms and they are summarized in Table 1. Of note, recent efforts in exome/genome-wide sequencing using massive parallel sequencing strategies of diverse human malignancies such as the TCGA (The Cancer Genome Atlas) have been completed and to date, mutations in Cxs and Panxs have not emerged as frequent events. Moreover, naturally occurring mutations have been reported in Cx genes and are frequently associated with complex disease phenotypes (Schalper et al., 2009). To our knowledge, no mutations in human Panx genes have been communicated. Taken together, this suggests that regulation of gap junction-type proteins in cancer cells could be more frequently associated with epigenetic and post-transcriptional events, as suggested in pioneering experiments characterizing mRNA transcripts using substractive hybridization methods in cultured normal and tumor breast epithelial cells (Lee et al., 1991).

**Table 1:** **Possible mechanisms responsible for reduced expression of connexins in human cancers and cell lines**.

**Protein**	**Tumor**	**Alteration**
Cx26	Squamous cell carcinoma oral cavity	Missense heterozygous single nucleotide mutation F142L (Rednam et al., 2011)
	Transitional carcinoma cell lines	Histone demethylation (Li et al., 2013)
	Small cell carcinoma (SCLC) and SCLC cell lines	Promoter hypermethylation (Chen et al., 2005)
	Invasive breast carcinoma (IBC) and IBC cell lines	Promoter hypermethylation (Tan et al., 2002)
Cx30	Colorectal carcinoma (CRC) and CRC cell lines	Promoter hypermethylation (Sirnes et al., 2011)
Cx32	Renal cell carcinoma cell lines	Promoter hypermethylation (Hirai et al., 2003)
	Gastric carcinoma	Promoter hypermethylation (Schalper et al., 2014, this report)
Cx36	Colorectal carcinoma (CRC) and CRC cell lines	Promoter hypermethylation (Sirnes et al., 2011)
Cx37	Colorectal carcinoma	Promoter hypermethylation (Sirnes et al., 2011)
	Hepatic angiosarcoma	High frequency of codon 319 polymorphism in vinyl chloride-related tumors (Saito et al., 2000)
Cx40.1	Hepatocellular carcinoma	Deletion (Zender et al., 2008)
Cx43	Colorectal carcinoma	Single nucleotide deletion A311V, Single nucleotide insertion I358N (Dubina et al., 2002)
	Non-small cell lung carcinoma	Promoter hypermethylation (Jinn and Inase, 2010)
	Prostate carcinoma cell lines	Histone deacetylation (Hernandez et al., 2006), miR-20a expression is inversely correlated with Cx43 expression (Li et al., 2012a,b)
	Glioblastoma multiforme cell line	miR-221/222 targets Cx43 mRNA, decreasing its expression (Hao et al., 2012)
	Nasopharyngeal carcinoma (NPC) and NPC cell line	miR-218 targets Cx43 mRNA and is downregulated in NPC (Alajez et al., 2011)
Cx45	Colorectal cancer cell lines	Promoter hypermethylation (Sirnes et al., 2011)
Cx62	Prostate carcinoma	6q14-21 deletion (Liu et al., 2007)

### Cx and Panx gene mutations in human cancer

Early efforts looking for Cx mutations in cancer specimens found that 4 of 7 primary human hepatic angiosarcomas carried a proline-to-serine substitution in codon 319 of the Cx37 gene (Saito et al., 2000). The same genetic variant was found in DNA extracts from adjacent non-tumor tissue, suggesting that it corresponded to a germline mutation or a single nucleotide polymorphism. The amino acid 319 is located in the cytoplasmic tail of the protein that contains diverse regulatory motifs. Of note, Cx37 has been shown to form functional hemichannels (Wong et al., 2006) and its expression reduced the proliferation of cultured rat insulinoma cells (Burt et al., 2008). In follow-up studies it was shown that introduction of Cx37 mutations that abrogate both the intercellular channel and hemichannel functions (T154A and C61A-C65A) failed to suppress the proliferation (Good et al., 2011, 2012). The inablity of the mutant protein to mimic the effect of the wild type Cx37 in cell proliferation indicates that the passage of molecules through Cx37-based channels is required to influence cell growth/survival. In addition, association between the C1019T polymorphism of the Cx37 gene, gastric adenocarcinomas and H. pylori infection was identified in a retrospective Chinese cohort (Jing et al., 2012).

Another study looking for Cx mutations in sporadic colorectal cancers found 2 frameshift Cx43 mutations in 3 of 6 studied tumors (a single nucleotide deletion A311V in 2 samples and a single nucleotide insertion I358N in 1 tumor) (Dubina et al., 2002). No alterations in Cx32 gene were identified. The Cx43 variants were mapped to the carboxy-terminal tail of the peptide and were not detected in DNA extracts from peripheral blood samples, confirming their somatic nature. However, such mutations have not been mechanistically characterized or further reported in larger tumor series.

Using a high-resolution array-based CGH platform to screen human hepatocellular carcinomas, deletions in the region encoding the Cx40.1 gene were identified (Zender et al., 2008). In support of a tumorigenic effect of this variant, an *in vivo*, xenograft-based RNAi screen showed that Cx40.1 downregulation using shRNA elicited a prominent acceleration of tumor growth. Little is known about the properties of human Cx40.1. For instance, it is expressed in the human heart (Söhl and Willecke, 2004), but its channel forming capacity remains to be proven.

Deletions of an 817 kb area at 6q14-21 containing the Cx62 gene were found in DNA extracts from 21 of 55 human prostatic adenocarcinomas using a high-resolution single nucleotide polymorphism array (Liu et al., 2007). However, the levels of Cx62 mRNA from the ONCOMINE database were comparable between tumor samples and normal controls, suggesting that another transcript located in this deleted segment such as MAP3K7 might have tumor suppressive actions.

Aggressive oral squamous cell carcinomas are predominantly associated with alcohol and tobacco use and are extremely rare in children. Interestingly, the case of a 6-year old girl was reported with psoriasiform dermatitis and sensorineural hearing loss that presented with a high grade, 3.3 cm palatal and nasal squamous cell carcinoma (Rednam et al., 2011). Due to the clinical findings the patient was screened and the single nucleotide Cx26 mutation F142L was found. Although there was no demonstration of causal relationship between the tumor and the Cx26 variant, other Cx26 mutations associated with excessive keratinocyte proliferation and syndromic hearing loss have been shown to produce hemichannels with increased function and altered permeability (Sánchez et al., 2010; Mese et al., 2011).

### Epigenetic regulation of expression of Cxs and Panxs in cancer

Epigenetic regulation of Cxs is a well-described process (reviewed in Vinken et al., 2009; Oyamada et al., 2013). Reduction in the expression of diverse Cxs with concomitant promoter hypermethylation has been shown in multiple human neoplasms and tumor cell lines. Downregulation of Cx32 gene expression by promoter hypermethylation was shown using bisulphite modified DNA-based PCR and a restriction enzyme-based assay in HK-2 and Caki-2 human renal cell carcinoma cells (Hirai et al., 2003). Treatment of cells with the demethylating agent 5-Aza-2′-Deoxycytidine restored Cx32 expression, confirming that Cx32 expression was suppressed. Similarly, downregulation of Cx43 in nasopharyngeal carcinoma CNE-1 cells was found to be associated with *GJA1* (Cx43) gene hypermethylation (Yi et al., 2007). A high frequency of Cx30, Cx36, and Cx37 promoter hypermethylation was also detected in colorectal carcinoma samples, as well as in Cx30, Cx36, and Cx45 in colon cancer cell lines (Sirnes et al., 2011). However, correlation between reduced transcript levels and the presence of promoter methylation was found only for Cx45. A more recent study identified Cx45 promoter hypermethylation in 33% of 485 colorectal carcinomas and a positive association with the actionable oncogenic BRAF exon 15 mutation (e.g., V600E) was noted (Ahmed et al., 2011).

**Figure 1:**
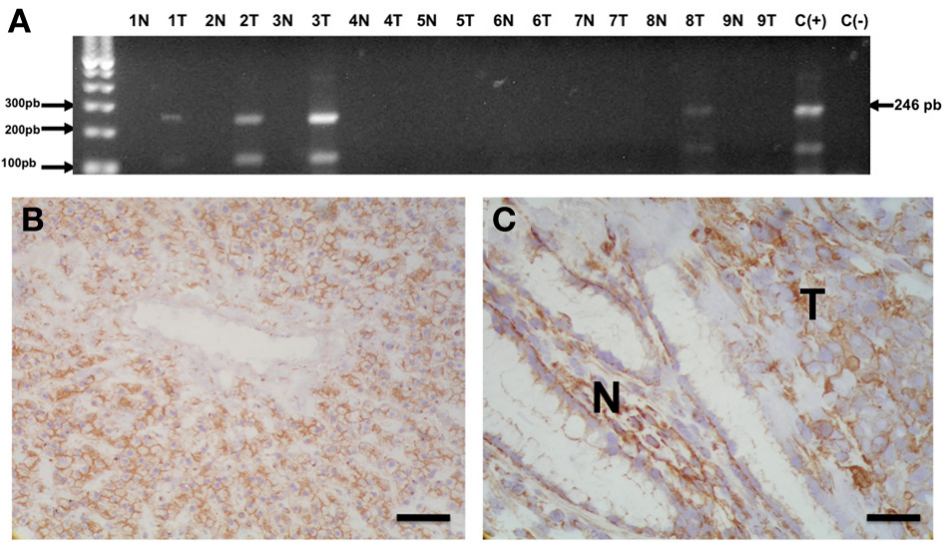
**Methylation of Cx32 promoter region in human gastric adenocarcinomas. (A)** Agarose gel showing the results from methylation specific PCR of sodium bisulphite-modified DNA extracted from 9 frozen gastric adenocarcinomas (1T-9T) and non-tumor tissue from the same subjects (1N-9N). In 4 cases, a 246 bp band corresponding to the methylated sequence in the Cx32 promoter region was identified. C(+) = Positive control sample of methylated DNA; C(−) = Negative control lacking template DNA. **(B,C)** Microphotographs showing Cx32 immunostaining in morphologically normal human liver **(B)** and non-tumor (N)/tumor (T) interface from one gastric adenocarcinoma sample **(C)**. Liver was used as positive control and showed homogenous and intense membranous-like staining. Cx32 immunoreactivity was also detected in the normal foveolar gastric epithelium (N) and in malignant epithelial carcinoma cells (T). The previously reported (Sánchez et al., 2009) monoclonal anti-Cx32 antibody clone 72F (dilution 1:1500, overnight incubation) was used to stain frozen sections. Preparations were then counterstained with hematoxilin. Bar = 100 μm.

Low Cx43 protein levels in human non-small cell carcinomas were associated with Cx43 CpG island promoter methylation and with heavy tobacco use (Jinn and Inase, 2010). Strikingly, the presence of Cx43 promoter hypermethylation in the non-tumor peritumoral lung tissue, but not in the tumor tissue proper, was significantly associated with lymph node positivity in non-small cell cancer patients (Chen et al., 2003). The biological determinants of this association are not clear. Reduced Cx26 protein and mRNA levels in a variety of lung carcinomas (including small-cell neuroendocrine carcinomas) and cell lines was also correlated with promoter hypermethylation and reverted by 5-Aza-2′-Deoxycytidine (Chen et al., 2005). Cx26 promoter hypermethylation was also found in 11 of 20 human invasive breast carcinomas and in cell lines (Tan et al., 2002). However, the absence of Cx26 expression in human squamous esophageal carcinoma cells was not correlated with the presence of Cx26 promoter hypermethylation, suggesting the presence of a different silencing mechanism (Loncarek et al., 2003). Using methylation specific PCR, we found Cx32 promoter hypermethylation in 4 of 9 frozen samples from human gastric adenocarcinomas (Schalper et al., unpublished observation, Figure 1). The 246 bp methylation-specific band was detected exclusively in the tumor and not in non-tumor areas of the samples (Figure 1A). However, we found no clear relationship between the presence of Cx32 promoter hypermethylation and Cx32 protein levels as detected by immunohistochemistry (Figures 1B,C). Taken together, these findings suggest that although more frequent in cancer tissues, promoter hypermethylation in Cxs genes is not unequivocally related with reduced/absent protein.

To our knowledge, regulation of Panx genes by epigenetic control has not been reported. Using the NIH PROSCAN webtool (http://www-bimas.cit.nih.gov/molbio/proscan/), we evaluated a 1000 bp region upstream of the initiation codon of the human Panx1 gene. We identified a 434 bp region with diverse transcription factor binding motifs and a significant probability of being a promoter region (Promoter score = 88.86; Promoter cutoff = 53.00). In the same genomic region and using the EMBL-EBI Cpgplot tool (https://www.ebi.ac.uk/Tools/seqstats/emboss_cpgplot/) we detected a 554 bp region with >50% C/G nucleotides, consistent with a CpG island. The latter suggests that human Panx1 has a CpG nucleotide-rich promoter region and could be modulated by hypermethylation.

Few events associated with histone modifications have been shown to affect Cx26 and Cx43 genes in human neoplasms. Li and collaborators (Li et al., 2013) recently found an inverse relationship between Cx26 and JARID1B (also known as KDM5B) histone demethylase protein levels in transitional cancer cell lines and advanced human bladder tumors. Overexpression of JARID1B was associated with reduced Cx26 protein in HT1376 and T24 cells, indicating that this demethylase represses Cx26 expression.

In androgen dependent (LNCaP) and androgen-independent human prostate cancer cell lines (DU145 and PC3), treatment with the histone deacetylase inhibitor Trichostatin A prominently increased Cx43 mRNA and protein expression. The intercellular transfer of the fluorescent dye Lucifer yellow was also increased by Trichostatin A, indicating increased gap junction mediated intercellular communication (Hernandez et al., 2006). Similar findings were observed in cultured non-malignant human peritoneal mesothelial cells (Ogawa et al., 2005).

### Post-transcriptional regulation of expression of Cxs and Panxs in cancer

Post-transcriptional modulation by small non-coding RNAs of Cx genes has also been reported in human tumors. Li et al. (2012a,b) found an inverse relationship between miR-20a and Cx43 protein and mRNA expression in human prostate tumors and cell lines. In this model, downregulation of miR-20a caused a 4-fold increase in Cx43 expression and reduced the cell proliferation. Using a luciferase reporter assay, the miR-20a antagomir increased the luciferase activity using the wild type Cx43 sequence, but was ineffective with Cx43 mutated at the miR-20a binding site at the 3′UTR region. The latter demonstrates that Cx43 is a direct target of miR-20a. Using a similar approach, miR-221/222 was shown to bind Cx43 mRNA, reduce Cx43 expression and promote cell growth and invasion in the human glioblastoma multiforme cell line U251 (Hao et al., 2012). Finally, transfection of miR-218 in nasopharyngeal carcinoma cells reduced Cx43 expression and was associated with increased apoptosis. Moreover, miR-218 transfection reduced the tumor growth after intramuscular implantation of C666-1 cells in SCID mice (Alajez et al., 2011). Although likely to occur, regulation of Cxs expression by lncRNAs has not been reported yet. In addition and to our knowledge, modulation of Panxs by non-coding RNAs has not been communicated.

## Hemichannels in cell proliferation and tumor progression

**Figure 2:**
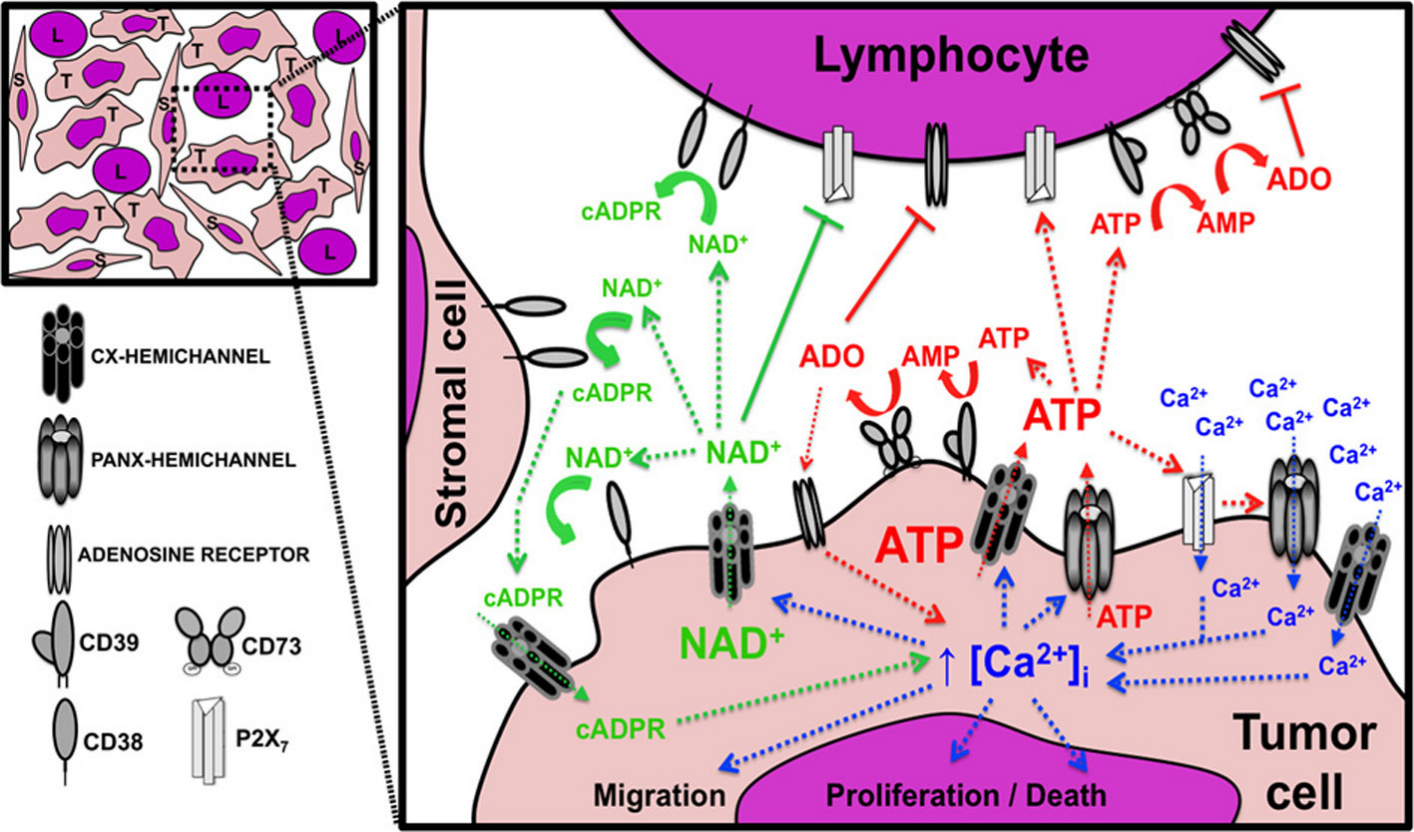
**Possible role of hemichannels in tumor growth and progression.** Diagram showing the paracrine interactions mediated by the passage of nucleotides and Ca^2+^ through hemichannels between tumor cells (T), lymphocytes (L), and stromal cells (S). The image in the upper left corner depicts the tumor microenvironment components with intimate relationship between the tumor and non-tumor cells. The expanded inset on the right represents a magnification of one tumor area and indicates the signaling circuits associated with NAD^+^ (green text and arrows), ATP (red text and arrows), and Ca^2+^ (blue text and arrows). The image also shows the presence and possible interactions between connexin (Cx) and pannexin (Panx) hemichannels, ectonucleotidases, purinergic receptors, and adenosine receptors. Arrows with dashed lines indicate a positive/stimulatory effect, arrows with continuous lines indicate a negative/inhibitory effect; and curved lines indicate (enzymatic) metabolic activity. ADO, adenosine; AMP, adenosine monophosphate; ATP, adenosine triphosphate; cADPR, cyclic adenosine diphosphate-ribose; [Ca^2+^]_i_, free intracellular Ca^2+^ concentration; NAD^+^, nicotinamide adenine dinucleotide; P2X_7_, P2X purinoreceptor 7. See text for detailed explanation of the biological consequences of each pathway.

The cellular consequences of altered hemichannel functions are believed to be mediated mainly by defective transmembrane transport of signaling molecules passing through them, leading to altered activation of intracellular pathways and autocrine/paracrine signals. Key molecules associated with cell proliferation/survival and shown to permeate activated hemichannels include NAD^+^ (nicotinamide adenine dinucleotide), cADPR (cyclic ADP-ribose), ATP, Ca^2+^, IP_3_, glutathione, and prostaglandin E_2_ (Wang et al., 2013a,b). However, additional, yet unidentified, relatively small-sized solutes could also mediate autocrine/paracrine effect in cells. In particular, the nucleotides NAD^+^ and ATP have been directly involved in hemichannel mediated cell proliferation (of non-tumor cells) by increasing the intracellular Ca^2+^ levels. Therefore, the exchange of these nucleotides and Ca^2+^ through hemichannels will be the main focus of this section. Also, the transmembrane passage of nucleotides constitutes a unique feeder system for extracellular nucleotidases that have a prominent role in the anti-tumor immune responses (see below). In addition, the increasingly recognized paracrine signaling between tumor and stromal cells that may occur through hemichannels could also support their involvement in tumor growth and cancer progression. A summary of the possible functions of hemichannels in cancer biology is presented in Figure 2.

### Hemichannels and NAD^+^ metabolism

The cellular NAD^+^ homeostasis is tightly regulated in all organisms and contributes to the maintenance of the energetic balance, intracellular redox potential and signal transduction. Tumor cells undergo metabolic adaptations to support growth and survival, including a pronounced shift from oxidative phosphorylation toward more NAD^+^ and lactate production through aerobic glycolysis, a phenomenon known as the Warburg effect (Chiarugi et al., 2012). The net cellular NAD^+^ content is the result from its synthesis by various enzymatic cascades involving niacin (vitamin B3), tryptophan, aspartic acid and reutilization of intracellular nicotinamide-related compounds, as well as NAD^+^ degradation through enzymatic hydrolysis, ribosylation and deacetylation. CD38 is the major mammalian NAD^+^ glycohydrolase and ADP-ribosyl cyclase, thus directly participating in NAD^+^ metabolism and production of second messengers (Chiarugi et al., 2012). However, the catalytic domain of CD38 is located in the extracellular domain, apart from its cognate intracellular substrate.

The first experimental evidence for the involvement of Cx43 hemichannels in transmembrane NAD^+^ transport came from studies using cultured NIH3T3 fibroblasts, HeLa cells and proteoliposomes (Bruzzone et al., 2001). In these models, NAD^+^ efflux required (and was paralleled by) Cx43 expression. Moreover, the NAD^+^ release was increased by lowering the extracellular Ca^2+^ concentration and prominently blocked by beta-glycyrrhetinic acid, La^3+^ and a Cx43 monoclonal antibody, thus implicating Cx43 hemichannels (Bruzzone et al., 2001). Shortly after, the same group convincingly demonstrated the involvement of Cx43 hemichannels in the [Ca^2+^]_i_-dependent, cADPR-induced cell proliferation in co-cultured mouse NIH/373 fibroblasts with or without CD38 expression (Franco et al., 2001; De Flora et al., 2004). In this model, the efflux of NAD^+^ through activated hemichannels allowed the interaction of this intracellularly produced dinucleotide with the extracellular catalytic segment of CD38, giving a mechanistic response to this topographical paradox. After its production by CD38-mediated NAD^+^ ribosylation, the ubiquitous second messenger cADPR can re-enter the cells to induce IP_3_-pathway independent-/ryanodine receptor induced Ca^2+^ transients and ultimately mediate cell cycling and proliferation. Later studies by another research group extended these observations and showed that cADPR re-uptake is mediated by Cx43 hemichannels through bidirectional NAD^+^ extrusion/cADPR import upon FCγ receptor stimulation in cultured murine J774A.1 macrophages (Song et al., 2011) (Figure 2, green colored arrows and text).

Although CD38 is expressed in various mammalian tissues including brain, prostate and muscle, it is most prominently found in lymphoid/blood cells (Deaglio et al., 2008). CD38 is frequently upregulated and has been indicated as a prominent oncogenic and adverse prognostic factor in hematologic malignancies including multiple myeloma and chronic lymphocytic leukemia. Moreover, humanized monoclonal antibodies targeting CD38 (e.g., daratumumab, MOR03087, and SAR650984) are under evaluation in early phase clinical trials to treat patients with advanced hematological B-cell malignancies (www.clinicaltrials.gov; trial identifiers NCT01615029, NCT00574288, NCT01749969, NCT01084252 and NCT01421186). As an integral component of the CD38-Cx43-cADPR axis and NAD^+^ degradation pathway, it is tempting to speculate that expression of Cx43 in neoplastic cells could serve as a prognostic/predictive biomarker for such compounds and modulation of Cx43 hemichannels may itself represent a novel anti-tumor therapeutic target, especially in CD38 expressing B cell malignancies.

Cx43 was shown to be expressed in isolated human B cells from tonsil and peripheral blood and Cx-based channels participate in immunoglobulin production and B cell maturation (Oviedo-Orta et al., 2000, 2001). In addition, Cx43 defective knockout mice have lower IgM-positive B cells than their wild type littermates and the defect is more pronounced in the homozygous than in the heterozygous animal, supporting a gene-dosage effect (Montecino-Rodriguez and Dorshkind, 2001). Although some authors have ascribed the functional effects of lymphocyte Cx43 to their participation in the pool of intercellular channels located at the cell-cell contacts between activated immune cells (e.g., “the immunological synapse”), the involvement of hemichannels in these processes has not been excluded. This is particularly relevant in immune cells that are expected to spend a considerable part of their lifespan detached from other cells. In this regard, the reduction in immunoglobulin levels observed by Oviedo-Orta and collaborators in primary human lymphocytes was triggered by traditional Cx-channel blockers and by a Cx43 mimetic peptide corresponding to the second extracellular loop (e.g., Gap27) (Oviedo-Orta et al., 2001), known to inhibit Cx43 hemichannels (Evans et al., 2006; Wang et al., 2012). Notably, additional short peptides corresponding to the intracellular domains of Cx43 (e.g., Gap18 and Gap20) were ineffective in this model.

More recently, Cx43 was shown to be required for activation and migration of cultured murine B cells (Machtaler et al., 2011). In this study, B cell responses were independent of intercellular gap junction channels and immunofluorescence experiments of Cx43 in the murine B lymphoma cell lines WEHI 231 and A20, and in primary murine B splenocytes, showed a predominant homogenous membranous distribution. The latter indicates a lack of preferential clustering/enrichment of Cx43 in areas of cell-cell contact. A similar diffuse membranous Cx43/CD38 co-localization staining pattern was observed in cultured J774A.1 macrophages (Song et al., 2011).

Surprisingly few studies have explored the expression of Cxs in native human lymphoid tissues and human lymphoid neoplasms. For instance, Cx43 was detected in germinal centers of fresh samples from human tonsil and spleen (Krenacs et al., 1997). Of note, Cx43 protein and mRNA were predominantly located in cells with irregular shape and co-expressing CD21 and CD35, consistent with follicular dendritic cells. Only scarce, more rounded cells with lymphocyte appearance and of undefined lineage were found to carry the Cx43 transcript in this study.

**Figure 3:**
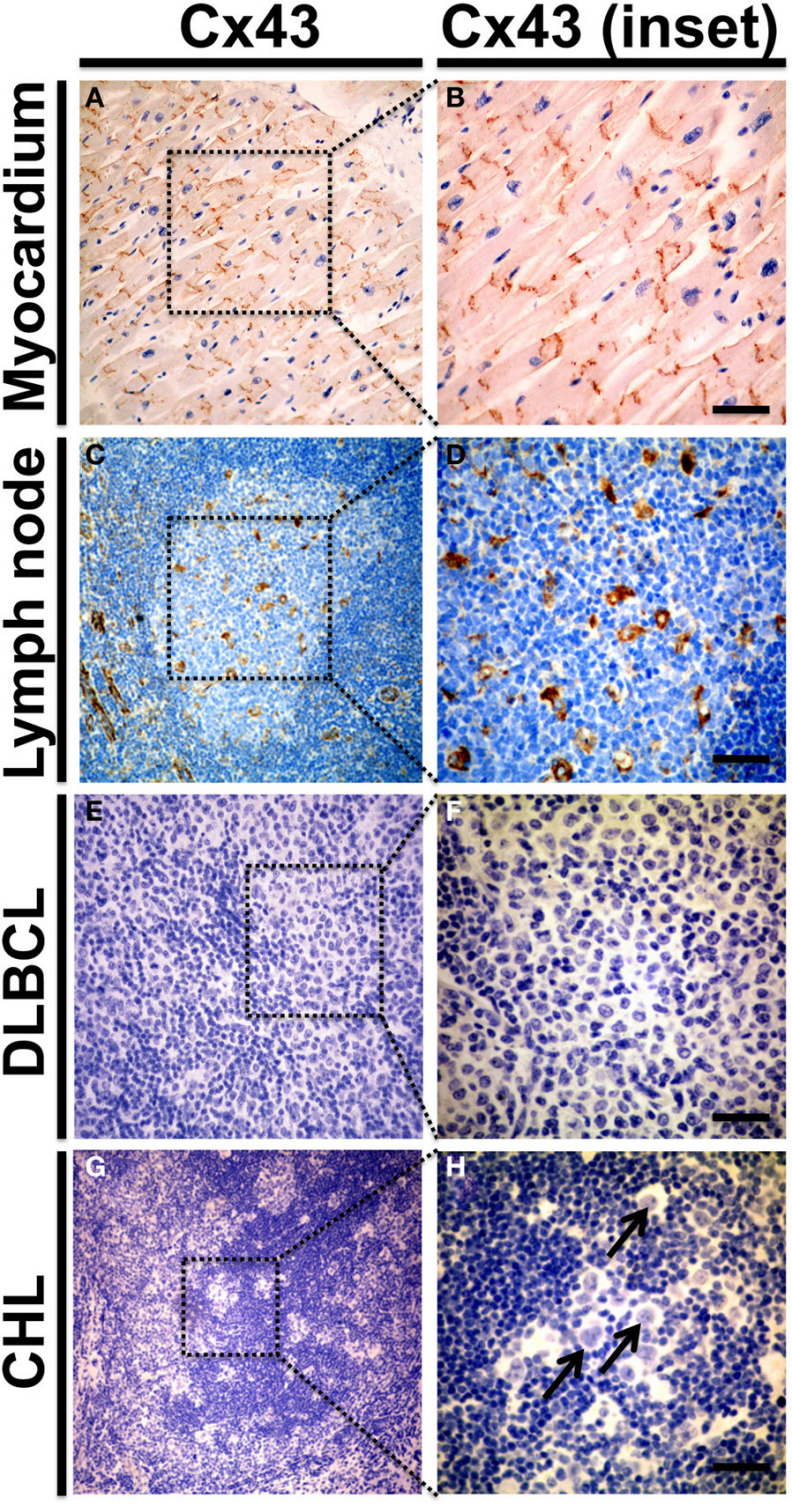
**Cx43 protein expression in human reactive lymph nodes and lymphomas.** Representative microphotographs from formalin fixed paraffin embedded samples of morphologically normal human myocardium **(***n* = 3, **A,B)**, reactive lymph node with follicular and parafollicular hyperplasia **(***n* = 4, **C,D)**, diffuse large B cell lymphomas **(***n* = 13 DLBCL, **E,F)** and classical Hodgkin's lymphoma **(***n* = 9 CHL, **G,H)**. Samples were included in a tissue microarray and stained with chromogenic immunohistochemical method for Cx43 using a mouse monoclonal antibody at 1:100 dilution (Zymed Laboratories). Preparations were counterstained with hematoxylin to visualize nuclei. Right panels **(B,D,F,H)** show magnified insets from low power images on the left **(A,C,E,G)**. **(A,B)** Depicts control Cx43 distribution in intercalated discs of human myocardiocytes. In non-tumor lymph nodes **(C,D)** Cx43 staining was predominantly detected in endothelial cells; and in cells with morphological features more consistent with macrophages and follicular dendritic cells. Cx43 was not detected in DLBCL and CHL samples **(E–H)**. Arrow in **(H)** indicates Hodgkin/Reed-Stemberg cells. Bar = 50 μm.

Aiming to characterize Cx43 expression in native human lymphoid tissues and lymphoid malignancies, preliminary experiments from our group using chromogenic immunohistochemistry detected low levels of Cx43 protein in formalin fixed-paraffin embedded samples from 4 non-tumor lymph nodes and absence of signal in a set of 22 malignant B cell lymphomas (13 Diffuse large B cell lymphomas [DLBCL] and 9 classical Hodgkin lymphomas [CHL]) (Figure 3). While distinctive Cx43-positive plaques were evident toward the intercalated discs of human myocardiocytes used as positive control (Figures 3A,B), only focal labeling of Cx43 in occasional germinal centers from reactive-type lymph nodes was seen in cells with morphology consistent with endothelial cells, macrophages and follicular dendritic cells (Figures 3C,D). The latter finding is somewhat consistent with previous observations by Krenacs and collaborators (Krenacs et al., 1997) and points to the possibility that Cx43 expression in lymphocytes might be too low to be detected by immunohistochemistry in unstimulated lymph nodes (e.g., lower than in myocardium). Alternatively, other Cxs known to be present in lymphocytes such as Cx40 (Oviedo-Orta et al., 2001) or Panxs could represent the main hemichannel forming pool in native human lymphoid tissues and lymphomas. Of note, Panx1 hemichannels have been previously shown to be expressed in T lymphocytes and to allow the passage of ATP that is structurally related with NAD^+^ and has a similar molecular mass (MW NAD^+^ = 663.42 vs. ATP = 551.14) (Woehrle et al., 2010). In addition, we found membranous-like Panx1 expression in samples from high grade, DLBCL that showed prominent co-localization with the pan-B cell marker CD20 in confocal stacks (Shoji et al., unpublished observation). Future studies using more sensitive methods and functional assays will be required to clarify this.

The presence of hemichannels in tumor cells even without CD38 expression might confer on them growth/migration advantages through increased autocrine/paracrine NAD^+^ signaling and the possible interaction with CD38 present in the membrane of adjacent non-tumor cells (e.g., stromal cells). In addition, relatively low increases in the extracellular NAD^+^ concentration (in the micromolar range) through cell death/lysis or release through activated hemichannels can exert local immune suppressive effects through activation of ionotropic P2X_7_ purinergic receptors in immune cells, leading to effector cytotoxic T cell death and expansion of immune suppressive regulatory CD4+/CD25+/FoxP3+ T cells (Tregs) (Scheuplein et al., 2009; Hubert et al., 2010). The latter effect could favor tumor progression by shielding cancer cells from immune-mediated cell killing (Figure 2). Indeed, increasing studies have found elevated Cx43 and/or Cx26 to be associated with more aggressive (e.g., shorter survival) and disseminated disease in diverse human malignancies and *in vitro* cancer models (Kanczuga-Koda et al., 2006; Tate et al., 2006; Naoi et al., 2007; Kyo et al., 2008; Cronier et al., 2009; Bui et al., 2011; Lamiche et al., 2012; Stoletov et al., 2013; Tang et al., 2013; Ghosh et al., 2014). In addition, a recent study demonstrated that increased post-treatment Cx43 levels were significantly associated with response to neoadjuvant chemotherapy in human breast carcinomas (Teleki et al., 2013) and high tumor Cx43 expression by immunohistochemistry was predictive of response to platinum-based chemotherapy in non-small cell lung cancer (Du et al., 2013). Notably, many of these studies reported predominant cytoplasmic location of Cxs and failed to detect a corresponding increase in the intercellular gap junctional communication. Recently available antibodies capable of selectively detecting (and blocking) Cx hemichannels through recognition of extracellular loop sequences (Riquelme et al., 2013a) might shed light on the relative contribution of hemichannels and intercellular channels in these responses.

### Hemichannels in ATP release and purinergic signaling

ATP is responsible for energy storage and transfer in the form of renewable high-energy phosphoryl bonds. In addition to its role in cell metabolism, intracellular ATP participates in the maintenance of electrochemical gradients through the fueling of pumps, active vesicular transport and also serves as a coenzyme and mediator for signal transduction and DNA synthesis. The cellular levels of ATP are determined by its constant production occurring mostly through oxidation of a variety of carbon-based substrates (e.g., aerobic cellular respiration); and degradation by hydrolysis into the lower level nucleotide phosphoforms ADP and AMP.

In addition to the prominent intracellular function, extracellular ATP serves as a pleiotropic intercellular messenger through its interaction with ionotropic (P2X_1_–P2X_7_) and metabotropic (P2Y_1_, P2Y_2_, P2Y_4_, P2Y_6_, P2Y_11_, P2Y_12_, P2Y_13_, and P2Y_14_) P2 purinergic membrane receptors (Falzoni et al., 2013). Examples of this include its function as neurotransmitter, proliferation stimulating agent, pro-inflammatory mediator and signaling factor between immune cells. In particular, extracellular ATP has been recognized to have a key role in the tumor-host interactions (reviewed in Di Virgilio, 2012). Despite its prominent extracellular role, the routes for cellular ATP release in both tumor and non-tumor cells are not well characterized. Diverse mechanisms have been proposed including passive transmembrane diffusion, vesicular release/exocytosis, cellular lysis and extrusion through specific chloride channels, ABC transporters, P2X_7_ receptors and Cx/Panx hemichannels (Di Virgilio, 2012; Baroja-Mazo et al., 2013; Falzoni et al., 2013; Orellana et al., 2013). The first direct indication of ATP release occurring through Cx hemichannels came from experiments showing that the propagation of Ca^2+^ waves in cultured astrocytes required the presence of extracellular ATP and Cxs expression, but not of functional intercellular channels (Cotrina et al., 1998). In addition, cultured C6, HeLa and U373 cells showed UTP-induced ATP release only after transfection with Cx26, 32, or 43 and the responses were mimicked by removal of extracellular Ca^2+^ (Cotrina et al., 1998). Later on, numerous studies confirmed the permeability of Cx hemichannels to ATP in diverse cell types and using an extensive repertoire of experimental conditions including the removal of extracellular divalents (Arcuino et al., 2002; Stout et al., 2002; Stout and Charles, 2003; Gomes et al., 2005; Pearson et al., 2005; Zhao et al., 2005; Bahima et al., 2006), mechanical stimulation (Arcuino et al., 2002; Stout et al., 2002; Gomes et al., 2005; Zhao et al., 2005; Richter et al., 2014), increased [Ca^2+^]_i_ (Braet et al., 2003a,b; De Vuyst et al., 2009), hypoxia/ischemic like conditions (Faigle et al., 2008; Clarke et al., 2009), membrane depolarization (Kang et al., 2008; Nualart-Marti et al., 2013), application of bacterial lypopolysaccharide (De Vuyst et al., 2007), treatment with amyloid beta peptide (Orellana et al., 2011), exposure to hypotonic stress (Lu et al., 2012), after spinal cord injury (Huang et al., 2012), in activated polymorphonuclear granulocytes (Eltzschig et al., 2006), induced by gamma irradiation (Ohshima et al., 2012) and after air-stimulation of cultured keratinocytes (Barr et al., 2013). Hemichannels formed by Panx1 have also been consistently shown to allow the passage of ATP in diverse cell types and experimental models (Bao et al., 2004; Locovei et al., 2006; Huang et al., 2007; Reigada et al., 2008; Schenk et al., 2008; Buvinic et al., 2009; Iglesias et al., 2009; Qiu and Dahl, 2009; Ransford et al., 2009; Chekeni et al., 2010; Garré et al., 2010; Murata et al., 2010; Sridharan et al., 2010; Woehrle et al., 2010; Seminario-Vidal et al., 2011; Dolmatova et al., 2012; Iglesias and Spray, 2012; Li et al., 2012a,b; Lohman et al., 2012; Sandilos et al., 2012; Xia et al., 2012; Xiao et al., 2012; Pinheiro et al., 2013; Riquelme et al., 2013b). Moreover, Panx1 hemichannels are functionally coupled to P2X and P2Y receptors activation (Locovei et al., 2006; Pelegrin and Surprenant, 2006; Wang et al., 2013a,b).

#### Hemichannels, ATP release and anti-tumor immune suppression

The levels of extracellular ATP correspond to the result between cellular release through diverse routes including hemichannels and degradation by ectonucleotidases (e.g., CD39 and CD73). Such enzymes mediate the extracellular conversion of ATP to AMP (CD39) and to adenosine (CD73) that interacts with predominantly immune inhibitory adenosine receptors (e.g., A2A), thus driving the shift from ATP driven pro-inflammatory environment to an adenosine-driven antiinflammatory/immune suppressive state (Antonioli et al., 2013) (Figure 2, red arrows and text). The latter mechanism might represent a negative regulatory switch to limit the inflammatory response and control tissue damage. However, relatively high concentrations of ATP (milimolar range) acting on P2X and P2Y receptors can induce marked suppressive effects in lymphocytes (Trabanelli et al., 2012; Burnstock and Di Virgilio, 2013). Consequently, increased extracellular ATP can potentially favor or suppress the local anti-tumor immune response, depending on its concentration, the presence and relative abundance of adenosine and P2 receptors in tumor and inflammatory cells, and the rate of conversion to adenosine by nucleotidases (Di Virgilio, 2012). In general, tumor microenvironments are considered to be purine-rich and CD39 and CD73 are commonly upregulated by hypoxia in diverse cancers leading to a more immune-suppressive tumor niche (Di Virgilio, 2012; Ghiringhelli et al., 2012; Antonioli et al., 2013). Consistent with this notion, expression of CD39 and CD73 are associated with more aggressive and metastatic tumors. The recent emergence of immunostimulatory drugs blocking immune inhibitory checkpoint pathways such as CTLA-4 and the PD-1/PD-L1 axis have shown unprecedented prominent and lasting clinical responses in patients with advanced solid tumors (Quezada and Peggs, 2013). Therefore, it is not surprising that strategies to inhibit CD39 and CD73 in cancer patients are being actively pursued. Unfortunately, most available *in vitro* and animal models to study the role of hemichannels in cancer cells are not helpful in evaluating their possible effect in the anti-tumor immune response (e.g., monocultures and immune defficient animals). Future studies using reconstituted cell culture systems including tumor cells and lymphocytes/APCs or immunocompetent animal models will help clarifying this issue.

#### Hemichannels, ATP release and cell proliferation

In addition to the inflammatory/immune effect, it has long been known that nucleotides have direct actions on tumor cells and most human neoplasms express a wide variety of purinergic receptors (Stagg and Smyth, 2010; Roger and Pelegrin, 2011; Burnstock and Di Virgilio, 2013). The effect of extracellular ATP in tumor cell proliferation/survival is complex and apparently depends on the ATP concentration, secretion pattern and the reportoir of purinergic receptors available in the target cell (Burnstock and Di Virgilio, 2013). Moreover, activation of the same receptor can induce apoptosis or even increase cell proliferation in different cell types. Extracellular nucleotides have been shown to induce anti-cancer effect in diverse tumor types and strategies to exploit this effect using ATP infusions or synthetic ATP analogs are ongoing. However, it is also clear that low doses of ATP as seen during spontaneous release from cells can have a pro-proliferative and growth promoting effect (Di Virgilio, 2012; Burnstock and Di Virgilio, 2013). In general, tumor cells have very high ATP content compared with non-tumor cells and strategies to reduce ATP have also been shown to enhance the activity of anti-cancer agents (Burnstock and Di Virgilio, 2013).

One of the most studied, yet still controversial P2 receptor in cancer cells is the so-called cytolytic P2X_7_ receptor. Although relatively high (millimolar range) concentrations of extracellular ATP can induce cell death through P2X_7_ receptor activation, more typically secreted (micromolar range) ATP concentrations are associated with promotion of cell survival/growth (Roger and Pelegrin, 2011; Di Virgilio, 2012). In support of this notion, expression of P2X_7_ receptor in some tumor models is associated with increased growth rate and metastases (Stagg and Smyth, 2010; Roger and Pelegrin, 2011; Di Virgilio, 2012). Also, signaling through some P2Y receptors such as P2Y_2_ has been associated with increased cell survival, proliferation and migration (White and Burnstock, 2006; Stagg and Smyth, 2010; Burnstock and Di Virgilio, 2013). In addition, it was recently shown that ATP released by non-tumor osteocytic cells treated with alendronate can either inhibit or stimulate the cell growth and migration of cultured MDA-MB231 breast cancer cell lines depending on the extracellular ATP concentration present (Zhou et al., 2014). While the inhibitory effect was seen with relatively low ATP concentrations and required P2X_7_ expression/activation; higher extracellular ATP levels were associated with increased tumor cell migration that was mediated by adenosine signaling through the A2A receptor. Similar observations were made in MDA-MB231 mouse xenographs, confirming the importance of this mechanism *in vivo*. Taken together, this indicates that both the release of ATP by adjecent non-tumor cells and the rate of conversion to adenosine participate in the ATP-mediated tumor cell proliferative responses. The biphasic effect of extracellular ATP could partially explain the dissimilar impact of ATP signaling in cell proliferation and survival seen in different models (Figure 2). Moreover, it suggests that treatment with non-hydrolizable forms of ATP could have prominent anti-cancer effects by limiting its conversion to pro-tumorigenic and immune supressive adenosine.

Notably, ATP release through activated Cx-hemichannels has been detected after stimulation with growth factors including nerve growth factor (NGF) (Belliveau et al., 2006); basic fibroblast growth factor (FGF-2) (De Vuyst et al., 2007) and acidic fibroblast growth factor (FGF-1) (Garré et al., 2010). NGF signaling has been implicated in diverse human malignancies (Molloy et al., 2011). In particular, NGF and its receptors are prominent oncogenic mediators in invasive human breast carcinomas and their expression in malignant cells is associated with adverse patient outcome (Dollé et al., 2004; Noh et al., 2013). Moreover, blockade of NGF signaling using monoclonal antibodies or reducing NGF expression with siRNA attenuates the proliferation and angiogenesis in MDA-MB-231 cells mouse xenografts (Adriaenssens et al., 2008). Aberrant FGFs signaling is also directly implicated in developmental tissue growth and in the progression of diverse human neoplasms and the genes encoding the four major FGF receptors are frequently amplified and/or mutated in human cancers (Dieci et al., 2013; Dienstmann et al., 2014). Research is ongoing to identify effective strategies to block FGF signaling using small molecule inhibitors and monoclonal antibodies to achieve anti-tumor effect with acceptable toxicity/safety profile. The aforementioned observations suggest that increased hemichannel activation possibly induced by excessive growth factors signaling could favor tumor cell proliferation and expansion. Supporting this possibility, Cx43 hemichannels were shown to mediate the proliferation of neural progenitor cells from the ventricular zone and the retinal epithelium through release of ATP, paracrine activation of purinergic P2Y receptors and subsequent [Ca^2+^]_i_ transients (Weissman et al., 2004; Pearson et al., 2005). In addition, two naturally occurring mutations in the human Cx30 gene (G11R and A88V) associated with a hyperproliferative keratinocytic genodermatosis termed hidrotic ectodermal dysplasia (or Clouston syndrome) have been demonstrated to produce defective Cx30 hemichannels with increased ATP release (Essenfelder et al., 2004). Although not directly measuring ATP, blockade of Cx43 hemichannels using the Gap26 mimetic peptide or carbenoxolone reduced the neointimal formation after vascular injury through decreased proliferation of smooth muscle cells (Song et al., 2009) and opening of Cx43 hemichannels mediated the survival signals induced by alendronate in osteocytic/osteoblastic cells through a Src-MAP kinases transduction dependent mechanism (Plotkin et al., 2002). The possible involvement of increased extracellular adenosine signaling in these responses has not been studied.

Similar to Cx hemichannels, ATP release through Panx1 hemichannels has been associated with increased proliferation of stem cells, neural progenitors (Wicki-Stordeur et al., 2012) and primary human cultured fibroblasts stimulated with histamine (Pinheiro et al., 2013). In the latter model, P2Y_1_ receptor-dependent [Ca^2+^]_i_ transients were triggered by Panx1-mediated ATP release. The release of ATP through activated Panx1 hemichannels was also shown to accelerate the assembly of multicellular C6 tumor cells aggregates in a 3D culture system (Bao et al., 2012). The latter effect was mediated by P2X_7_ receptors activation and remodeling of the F-actin cytoskeleton, and ultimately suggests that Panx1 could particiapte in tumor cell motility.

Consistent with a pro-tumorigenic effect, Panx1 (but not other Panxs) was found at relatively high levels in the melanoma cell lines B16 and was associated with membranous-like location and increased hemichannel function (Peñuela et al., 2012). In this model, down regulation of Panx1 using siRNAs significantly reduced the expression of the malignant melanoma markers vimentin, hsp70 and B-catenin, and diminished the cell density and motility, but increased melanin production suggesting acquisition of a more differentiated phenotype. Moreover, Panx1 silencing reduced the tumor size and the frequency of liver metastases in a chicken embryo tumor implantation model, suggesting that Panx1 hemichannels favor melanoma growth and progression (Peñuela et al., 2012). Similarly, silencing of Panx2 induced the acquisition of a more differentiated neuronal-like phenotype in N2A neuroblastoma cells (Swayne et al., 2010). Therefore, it would not be surprising to find specific tumor types that use hemichannels mediated mechanisms for their growth and dissemination. Also, the presence of subpopulations of cells with activated hemichannels, and clones with closed hemichannels or even lacking hemichannels in their membranes within a given tumor is possible. Dynamic adaptations of tumor cells to environmental pressure could also support the opportunistic transit between the functional states of hemichannels in tumor cells (e.g., proliferation vs. migration or invasion).

#### Anti-tumor effects of ATP release through hemichannels

**Figure 4:**
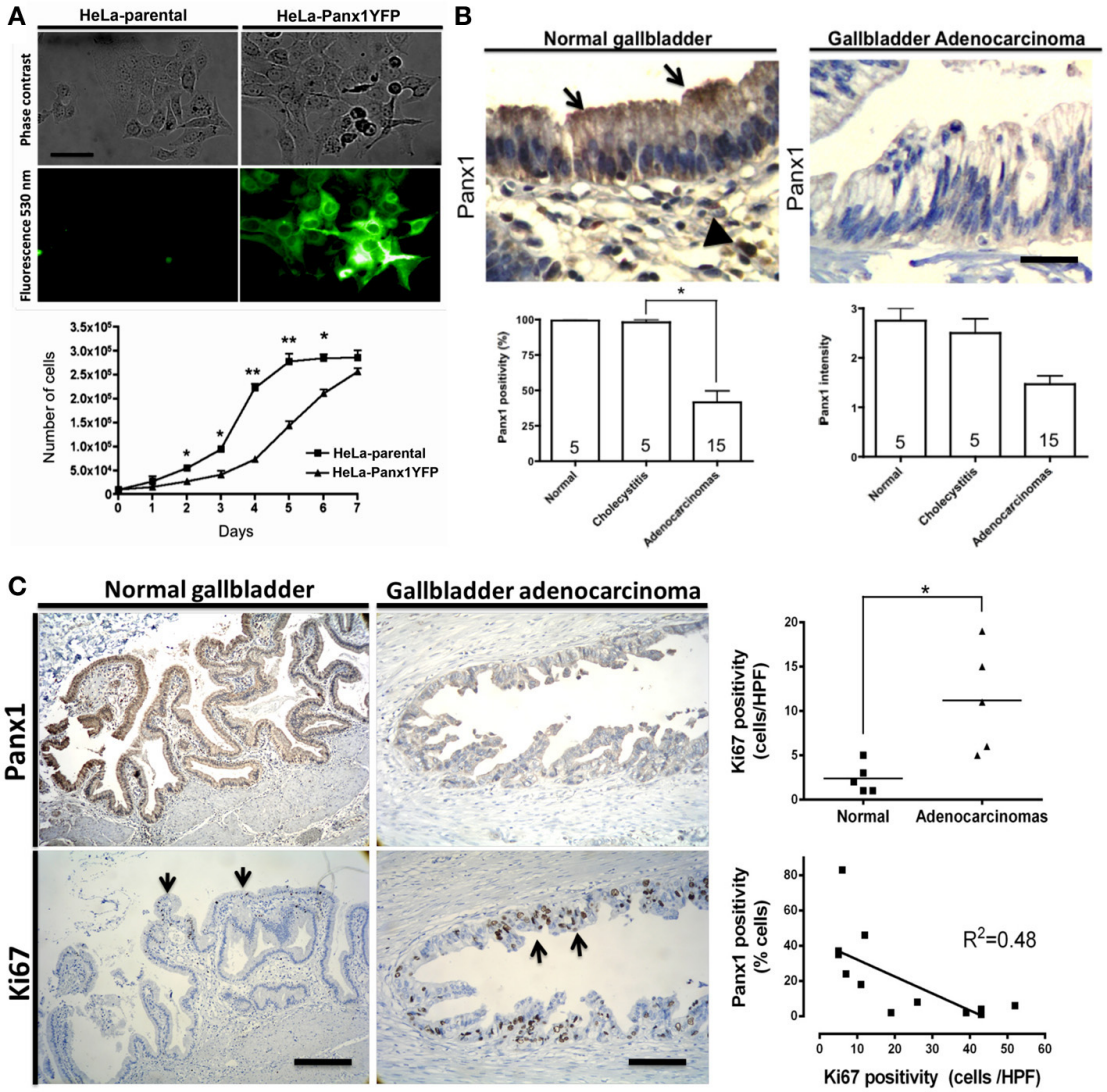
**(A)** Exogenous Panx1 expression reduces cell density in cultured HeLa cells. Microphotograph showing phase contrast (upper panel) and fluorescence images (lower panel) of cultured parental HeLa cells (left) and HeLa cells stably expressing Panx1-YFP (right, green channel 530 nm). Graph showing the cell density of parental (boxes) and Panx1-expressing HeLa cells (triangles) at different days of culture. Cells were exposed to 10% fetal bovine serum and counted each 24 h for 7 days. Mean ± s.e.m. of 6 independent experiments is shown. Bar = 30 μM. **(B)** Panx1 expression is lower in gallbladder adenocarcinomas. Upper-left panel: Immunohistochemical Panx1 expression using a previously reported antibody (Buvinic et al., 2009) in morphologically normal gallbladder showing positivity in epithelial cells (arrows) and mononuclear inflammatory cells from the lamina propria (arrowheads). Upper-right panel: Panx1 expression in gallbladder adenocarcinoma showing lower Panx1 expression in tumor cells. Lower panels: Panx1 expression (left) and signal intensity (right) in normal gallbladders, chronic cholecystitis, and gallbladder adenocarcinomas. The amount of positive cells is expressed as percentage ± s.e.m. Intensity is expressed as arbitrary levels (1: weak; 2: moderate; 3: strong). Experimental conditions remained the same in all experiments. The number of cases in each category is indicated within the bars. Bar = 25 μm **(C)** Panx1 expression is inversely related with proliferation in gallbladder carcinomas. Representative microphotographs showing immunohistochemical Panx1 signal (upper panels) and Ki67 nuclear staining (lower panels, arrows) in normal gallbladder (left) and in gallbladder adenocarcinoma (right). Graph showing the Ki67 index in normal samples (black boxes) and in carcinomas (triangles). Linear regression analysis showing inverse relationship between Panx1 and Ki67 positivity in gallbladder carcinomas. *R*^2^ = 0.48; ^*^p < 0.05 and ^**^p < 0.01. Bar = 400 μm.

Although a good deal of evidence points to the possible pro-tumorigenic and proliferative effect of both Cx and Panx1 hemichannels activation, it is relevant to consider that association with reduced cell proliferation/survival is also apparent. Moreover, sustained or de-regulated opening of hemichannels can be deleterious for cells. In fact, activation of Cx43 or Panx1 hemichannels by noxious stimuli such as ischemic-like conditions or oxidative stress can accelerate self and paracrine cell death through massive ATP-release and [Ca^2+^]_i_-related mechanisms (Contreras et al., 2004; Retamal et al., 2007b; Decrock et al., 2011; Carette et al., 2014) (Figure 2). Consistent with an anti-tumor effect, exogenous Panx1 expression was associated with decreased proliferation, motility and anchorage-independent growth in cultured C6 glioma cells (Lai et al., 2007). Similar findings were obtained after expression of Panx2 (Lai et al., 2009). In addition, Panx1 and Panx2 expression were associated with reduced tumor volume in murine C6 tumor implants, which led to propose them as tumor suppressor genes. In line with this observation, forced expression of Panx1 and Panx3 increased the dye uptake and reduced the proliferation of cultured rat epidermal keratinocytes (Celetti et al., 2010). Data from our group revealed that exogenous Panx1 expression significantly reduced the cell density of cultured HeLa cells (Figure 4A). In addition and using a previously reported antibody (Buvinic et al., 2009), we found that Panx1 is present in morphologically normal human gallbladder epithelium and its levels are lower in gallbladder adenocarcinomas (Figure 4B). Also, in gallbladder tumors Panx1 signal is inversely related to the proliferative activity as determined by the nuclear Ki-67 positivity rate (Figure 4C). Of note, the rate of nuclear Ki-67 positive cells is frequently used as a metric of the tumor proliferation grade and has prognostic value in diverse human malignancies such as gliomas, breast carcinomas and melanomas (Weigel and Dowsett, 2010).

The apparent discordances between the effects of Panx1 expression in proliferation and motility of melanoma, glioma and epithelial tumor cells could be due to tumor cell-specific and/or cell lineage-specific functions of hemichannels. Distinct alterations in oncogenic pathways between melanocytic, glial and epithelial tumors are well known.

### Hemichannels in intracellular Ca^2+^ balance, cell survival and migration

Intracellular Ca^2+^ is recognized as key second messenger and master regulator of cell fate. Altered cytosolic Ca^2+^ can be associated with cell death and cell proliferation, depending on the cell type, concentration and biological context. The intracellular Ca^2+^ balance is tightly regulated and active mechanisms have evolved to keep its levels in the low nanomolar range under resting conditions. The extracellular levels of Ca^2+^ are several orders of magnitude higher (e.g., milimolar range), creating a prominent inward concentration gradient. A complex signaling system administrates dynamic intracellular Ca^2+^ reservoirs within organelles such as the endoplasmic reticulum, mainly through the function of endomembranous ion channels, Ca^2+^ ATPases and binding to Ca^2+^ rich proteins (Berridge et al., 2003). Also, this system allows the functional coupling between intracellular Ca^2+^ stores and extracellular signals through activation of plasma membrane receptors. Such surface receptors include the dimeric tyrosine kinase growth factor-type receptors (e.g., EGFR, FGFR, PDGFR, etc.) and a wide array of seven-transmembrane domain G-protein coupled receptors (e.g., P2Y purinergic receptors, peptide hormone receptors, chemokine receptors, neurotransmitters, etc.). In addition, the influx of Ca^2+^ through ligand-activated channels also participates in the intracellular Ca^2+^ homeostasis. Intracellular Ca^2+^ signaling and the generation of intercellular Ca^2+^ waves have been involved in key cancer-related process including escape from apoptosis, perpetuation of growth, cell migration, metastasis and promotion of angiogenesis (Monteith et al., 2007; Parkash and Asotra, 2010; Monteith et al., 2012). In particular, Ca^2+^ increases have been shown to favor cell proliferation through altered transcription and enhance cell motility through remodeling of actin cytoskeleton and focal adhesions (Lee et al., 2011; Monteith et al., 2012). Moreover, remodeling of Ca^2+^ signaling is a feature of diverse cancers and consists frequently in altered expression and function of Ca^2+^ channels and pumps (Roderick and Cook, 2008; Monteith et al., 2012). For instance, purinergic receptors, TRP channels, store operated Ca^2+^ channels (e.g., ORAI and STIM) and Ca^2+^ pumps are deregulated in diverse human tumors (Lee et al., 2011; Chen et al., 2013). Intracellular effectors commonly involved in the pro-oncogenic effects of Ca^2+^ include protein kinases (e.g., PKC), phosphatases (e.g., calcineurin), transcription factors (e.g., NFAT) and Ca^2+^-sensitive signaling proteins (e.g., calmodulin).

As discussed, the activation of hemichannels is linked to the intracellular Ca^2+^ balance by mediating the autocrine/paracrine signaling through transmembrane exchange of nucleotides and Ca^2+^-modifying second messengers (reviewed in Wang et al., 2013a,b) (Figure 2, blue arrows and text). In addition, early observations suggested that hemichannels activated by metabolic inhibition induced massive Na^+^ and Ca^2+^ entry in cultured rabbit myocardiocytes (Li et al., 2001). In support of this notion, recent data using reconstituted systems support that hemichannels formed by Cx26 (Fiori et al., 2012), Cx32 (Sánchez et al., 2009) and Cx43 (Schalper et al., 2010) allow Ca^2+^ influx (reviewed in Orellana et al., 2012). Notably, increasing the [Ca^2+^]_i_ with endogenous ligands (e.g., FGFs) or a Ca^2+^ ionophore enhances the activation of hemichannels formed by Cx32 and Cx43 (De Vuyst et al., 2006, 2009; Schalper et al., 2008a; Garré et al., 2010). Also, autocrine/paracrine signaling by additional (non-nucleotide) hemichannels permeants such as IP_3_, glutamate and prostaglandins involve increased intracellular Ca^2+^ (Schwartz and Alford, 2000; Meves, 2006; Gossman and Zhao, 2008; Traynelis et al., 2010), suggesting the possibility of hemichannels-mediated Ca^2+^ influx/Ca^2+^-increase/Ca^2+^ influx loops (see Figure 2). To our knowledge, Panx hemichannels have not yet been proven to be Ca^2+^ permeable. However, they can also be activated by increased [Ca^2+^]_i_ and the permeability properties of Panx1 hemichannels suggest that they are likely to allow the passage of ions. Taken together, these data suggest that under specific pro-tumorigenic circumstances (e.g., altered signal transduction and tissue microenvironment) activated hemichannels could favor cancer progression by directly increasing intracellular Ca^2+^-dependent tumor cell proliferation and motility.

## Conclusions and future perspectives

Cxs and Panxs are frequently altered in human tumors and cell lines. Emerging data has challenged the notion that these proteins are tumor suppressors and suggest that they could favor tumor growth and dissemination in some circumstances. Although diverse technical aspects limit the study of hemichannels, current data support their involvement in the modulation of cell proliferation and migration. The presence, regulation and characteristics of hemichannel functions in cancer cells remain largely unexplored, but their permeability to nucleotides and Ca^2+^ suggest a possible role in favoring tumor growth and disease progression. Future efforts to characterize the expression and specific functions of hemichannels in diverse human malignancies will support their use as prognostic/predictive markers and to design novel anti-cancer therapeutic strategies.

### Conflict of interest statement

The authors declare that the research was conducted in the absence of any commercial or financial relationships that could be construed as a potential conflict of interest.
